# Towards a Comprehensive Strategy for the Management of Rare Diseases in Slovenia: Outlining an IT-Enabled Ecosystemic Approach

**DOI:** 10.3390/ijerph182312395

**Published:** 2021-11-25

**Authors:** Dalibor Stanimirovic, Eva Murko, Tadej Battelino, Urh Groselj, Mojca Zerjav Tansek

**Affiliations:** 1 National Institute of Public Health, Trubarjeva 2, 1000 Ljubljana, Slovenia; eva.murko@nijz.si; 2 Department of Endocrinology, Diabetes and Metabolism, University Children’s Hospital Ljubljana, Bohoriceva ulica 20, 1000 Ljubljana, Slovenia; tadej.battelino@mf.uni-lj.si (T.B.); urh.groselj@kclj.si (U.G.); mojca.zerjav-tansek@mf.uni-lj.si (M.Z.T.); 3 Faculty of Medicine, University of Ljubljana, Vrazov trg 2, 1000 Ljubljana, Slovenia

**Keywords:** rare diseases, patient treatment, ecosystemic approach, case study, Slovenia

## Abstract

Rare diseases (RDs), with distinctive and complex features, pose a serious public health concern and represent a considerable challenge for the Slovenian healthcare system. One of the potential approaches to tackling this problem and treating patients with RDs in a quality and effective manner is to form an RD ecosystem. This represents a functional environment that integrates all stakeholders, procedures, and relationships required for the coordinated and effective treatment of patients. This paper explores the current situation in the field of RDs, especially in light of the proposed ecosystemic arrangement, and provides an outline for the design of an RD ecosystem in Slovenia. The research applies a case-study design, where focus groups are used to collect evidence from the field, assess the state of affairs, and generate ideas. Structured focus group discussions were conducted with preeminent experts affiliated with the leading institutions in the field of RDs in Slovenia. Analyses and interpretations of the obtained data were carried out by means of conventional content analysis. Setting up an RD ecosystem in Slovenia would lead to significant benefits for patients, as it could promote the coordination of healthcare treatment and facilitate extensive monitoring of the treatment parameters and outcomes. A well-organized RD ecosystem could garner considerable systemic benefits for evidence-informed policymaking, a better utilization of resources, and technological innovation. Delivering quality healthcare in this complex field is largely reliant on the effective integration and collaboration of all entities within the RD ecosystem, the alignment of related systemic factors, and the direction of healthcare services to support the needs and well-being of patients with RDs.

## 1. Introduction

According to some analytical estimates, there are approximately 150,000 patients with RDs in Slovenia [1,2]. Notwithstanding the paucity of reliable epidemiological data, the outlined numbers indicate that this field is of major concern for the Slovenian healthcare system [2], which remains considerably underfinanced across various levels, and continuously incapacitated in terms of healthcare resources [3]. Substantial problems persist in relation to operating efficiency, increasing costs, and long waiting periods for some healthcare services [4,5]. In addition to a deficiency of consistent epidemiological evidence and the overall lack of understanding and resources in the field, RDs commonly have intricate characteristics, which additionally intensify the gravity of this matter for the Slovenian healthcare system [6]. In 80% of cases, they are of a genetic origin; in 70% of cases they affect children; and they are often incurable, advancing and life-threatening [1]. It is discernible that the wide-ranging difficulties confronted by healthcare systems in handling RDs extend to several factors, which pose major challenges for national governments [7]. The integration of these factors in a new intelligible and efficient structure necessitates innovative changes to the current setting in the field of RDs. Newly established and innovative structures in different sectors, which comprise human stakeholders and the relevant tangible and intangible components, as well as their interrelationships, are progressively being dubbed *ecosystems* [8]. The ideal ecosystem in the field of RDs should therefore provide a functional environment that connects all policy and normative facilitators, institutional stakeholders, and the IT-enabled instruments required for the organized and comprehensive medical treatment of patients with RDs. An IT-enabled RD ecosystem must contain a regulatory framework, government agencies, insurance institutions, healthcare treatment and rehabilitation providers, patient and professional associations, national and supra/international RD organizations, IT tools and platforms (the National RD Registry and the National Contact Point for RDs (NCP)), the academic and research community, and operational mechanisms between all the listed entities, based on streamlined business processes and clear rules of operation [1].

A large number of the itemized components from the favored ecosystem in the field of RDs are already in place in Slovenia and operate effectively (also on an international scale), however, some of these components are rather inactive and isolated, thus missing opportunities for more effective cooperation and performance. As a result, the whole field of RDs is affected by developmental challenges, coordination issues, and high operational expenses, as well as an inability to capitalize on existing institutional potentials and organizational synergies to address the expanding and increasingly diversified demands of RD patients [4,9]. Given this situation, where essential components are not properly connected and often work incoherently, it is not possible to utilize all healthcare system capacities, which ultimately impairs the process of treating patients with RDs, on the one hand, and inevitably causes the irrational spending of already insufficient resources in healthcare, on the other. In order to develop a truly beneficial ecosystem in the field of RDs, it is necessary to set up an adequate normative framework, integrate all these components, manage their operation, and mitigate the problems associated with the coordination of stakeholders and the lack of resources. The integration of ecosystem components can only be realized by appropriate leveraging of IT solutions, which should act as the main facilitator for the establishment of a collaborative multicomponent RD ecosystem, that includes operational relations, which are materialized in optimized business processes, and advanced organizational enhancements [10,11]. In view thereof, the aim of this paper is to explore the existing state of affairs concerning RDs and to outline the potential development directions in the field, especially in light of the proposed establishment of the RD ecosystem in Slovenia. Accordingly, this paper primarily focuses on outlining an ecosystemic approach to managing RDs in Slovenia.

## 2. Materials and Methods

### 2.1. Research Design

The research design was initially prepared for the development and implementation of the individual key elements in the field of RDs, such as the National RD Registry. However, during the preparatory stages of the research we found that the problem relating to RDs is much broader, and reaches beyond individual elements, and as such requires a much more comprehensive approach in terms of designing a plan and outlining the structure of a functional and wide-ranging ecosystem in the field of RDs. In view of this, the paper applies a case-study design to explore the defined research objectives concerning the potential ecosystemic approach to managing RDs in Slovenia. The research was intended to be exploratory in character, since the intricate field of RDs in Slovenia is still fairly underdeveloped. Therefore, quantitative evidence from the field was not available, which could compromise the credibility of the research results. Accordingly, a qualitative methodological approach was deemed more advantageous considering the specificities of the research area [12,13], and focus groups were conducted for data collection (14 sessions from January 2016 to February 2017). 

### 2.2. Sample

Participants in the focus groups were selected for their expertise and experience relating to RDs and their systemic management. The proficiency and integrity of the focus group participants were crucial for their productive participation in the study process and the overall credibility of the study. Through a non-random stratified sampling method and after reaching the saturation point, a representative sample of 24 healthcare experts was curated. Interest in participating in the study was high and all of the experts who were contacted participated in the focus groups—the participation rate was 100%. The participating experts were affiliated with leading institutions in the country in the field of RDs, including the University Medical Center Ljubljana (UMCL)/University of Ljubljana, Faculty of Medicine (11 experts), the National Institute of Public Health (3 experts), the University Medical Center Maribor/the University of Maribor, Faculty of Medicine (6 experts), the Slovenj Gradec General Hospital (2 experts), and IT companies (2 experts). The participating healthcare professionals and IT experts were specialized in the areas pertinent to RDs and health information systems that are required for the successful introduction of an IT-enabled ecosystemic approach. More detailed characteristics of the sample and the areas of work and research of the participating experts are provided in the table below (Table 1).

### 2.3. Data Collection

Before conducting the focus group sessions, the participants were once again carefully informed of the purpose and objectives of the study. Following the constructive suggestions of the participating experts, some additional clarifications were made in order to resolve potential uncertainties and misconceptions. UMCL made the arrangements for the focus group sessions in terms of providing space and logistics. The controlled discussions typically lasted around 120 min and were documented in writing. They concentrated on the state of affairs in the field of RDs, the main concerns, the required priority actions, and the critical aspects, whether at the policy/normative, institutional/organizational, or technological/data management levels, for the potential introduction of an ecosystemic approach to the management of RDs in Slovenia. The focus group participants performed a comprehensive analysis of the field, identified key strategic directions, and subsequently outlined a potential ecosystemic approach that could be applied for the management of RDs in Slovenia.

### 2.4. Data Analysis

The data were analyzed and interpreted using conventional content analysis techniques, while the outline of the ecosystemic approach was derived from the focus group discussions and literature review [1,2,6,7,8,9,10,11,14,15,16,17]. The content analysis involves the coding of the key claims associated with distinct constructs highlighted by the focus group participants [18,19]. Discussions between the focus group participants served as a platform for defining the key coding categories [20]. In order to enhance the integrity and validity of the conclusions reached [21], a concluding content analysis was conducted independently by different authors. Following the data analysis, a subsequent outline of the ecosystemic approach and charts representing its essential components were obtained in collaboration with the participating experts, who played a constructive role throughout all phases of the study. The current situation regarding RDs in Slovenia was comprehensively examined after an exhaustive study of the literature and an analysis of different sources covering RDs and ecosystem-related information. In the following phase, the research was particularly focused on efforts to provide a feasible platform for the potential introduction of the ecosystemic approach in the field of RDs in Slovenia. The experts participating in the study played a dual role. Initially, they were required to take part in an assessment of the current settings and to identify the critical aspects in the field of RDs. Additionally, using their unique insight and their knowledge of the field, they were asked to provide starting-points for the proposed ecosystemic approach for the management of RDs in Slovenia.

## 3. Results

The experts participating in the focus groups outlined the state of affairs in the field and unanimously agreed on which critical aspects should be analyzed at the outset of the study (Table 2). Focus group participants underlined that these starting points should be taken into account in further steps and the gradual design of the ecosystemic approach.

### 3.1. A Cross Section of the Critical Aspects in the Field

#### 3.1.1. Policy and Normative Aspects

In order to successfully establish an RD ecosystem, certain elementary preconditions must be met. Policy orientations and legal bases represent a *conditio sine qua non* of the successful implementation of an RD ecosystem. The absence of a long-term strategic framework and the lack of current operational policies pose serious problems in the field of RDs, reflected in all connecting areas, leading to negative impacts on all related factors in the existing system in the field of RDs. The last nationally adopted legal framework in the field is the “Work Plan in the Field of Rare Diseases in the Republic of Slovenia” [1], which expired last year. Regrettably, an up-to-date strategic document and action plan for the future have not yet been prepared. The intermittent interest and a lack of healthcare policy commitment in the field generally result in insufficient funding and a poor engagement of the stakeholders. Such conditions typically undermine all efforts to establish such an ecosystem. 

As part of recent activities in the field, the 2-year project “Analysis and development in the field of rare diseases in Slovenia” was initiated in October 2015. The project objectives focused on the overall analysis of RDs in Slovenia, the documentation of systemic constraints in the field, and on defining the structure of the pilot National RD Registry [2]. Although the project yielded some noticeable and applicable results (e.g., the design of the pilot National RD Registry), the further development of the RD ecosystem remains incomplete, and urgently requires further review.

As far as the national normative framework is concerned, the valid Healthcare Databases Act was adopted at the end of April 2018 [22], after a long period of legislative gridlock. The adoption of the amended Act is crucial for the desired rearrangements to be enacted in the field, as it ensures the mandatory legal bases for processing data on RD patients and for the implementation of the national RD ecosystem.

In order to apply an IT-enabled ecosystemic approach, the strategic and regulatory framework should include more detailed financial and operational plans with the projection of staff and equipment resources as declared in the “Work Plan in the Field of Rare Diseases in the Republic of Slovenia 2010–2020”. The roles of the main stakeholders should be defined more precisely, as well as the financial and work timelines. The competent group at the governmental level should be held accountable for the realization of the plan and the coordination of healthcare providers.

#### 3.1.2. Institutional and Organizational Aspects 

Medical specialists and professionals in laboratory medicine have very limited resources for work relating to RDs in a small nation of two million inhabitants, also taking into account the low number of patients with a specific diagnosis, which makes the organization of RD patient associations much more problematic. The IT-enabled RD ecosystem needs to promote the continuous education and recruitment of medical and other specialists and provide a good plan for the inclusion of patients with many different diagnoses in a well-organized and supportive environment.

The number of institutions dealing with RDs in Slovenia is relatively small. Patients with RDs are, in most cases, treated and rehabilitated at five institutions—the UMCL, the Golnik University Clinic of Respiratory and Allergic Diseases, the Slovenj Gradec General Hospital, the Valdoltra Orthopedic Hospital, and the University Rehabilitation Institute (Soča). Despite the relatively low number of institutions working in the field of RDs, there are opportunities for organizational improvements, especially in terms of coding processes, better communication, the utilization of institutional capacities and resources, the coordination and streamlining of work processes, and cooperation in planning the continuous treatment of RD patients.

The coding of RDs in Slovenia is generally based on established national practices and field particularities. The rather constricted listing of dedicated codes for RDs included in the International Statistical Classification of Diseases and Related Health Problems (ICD-10-AM, Version 6) for approximately 6000 RDs contributes significantly to the inconsistencies and heterogeneity of the coding process [2]. The 11th revision of the ICD was released on 18 June 2018 and is greatly improved in relation to the field of RDs [23]. It contains, thus far, approximately 5400 specific codes for RDs; however, the implementation process for this version in Slovenia will certainly take a few years. The appropriate coding of RDs is required as soon as practicably possible. Additional coding with ORPHAcodes (unique and stable identifiers for RDs) will prove helpful in all follow-up stages, in the planning of treatments and resources, alongside its inclusion in the expected National RD Registry. It is true that ICD-11 will improve the situation, but the rapidly increasing number of new RDs require additional international coding. Such coding should be a part of the Slovenian eHealth databases and should be made available to all healthcare institutions.

While trying to establish a convenient ecosystem for the management of RDs, an applicable model of organization and sustainable funding must be considered in the early planning stages. Ensuring suitable resources is a key prerequisite for the effective development of a national project of this sort, and its successful implementation in the complex healthcare environment. Moreover, the potential of the successful management of RDs will largely depend on the resources (financial, human, IT, organizational) available for the day-to-day operation of all institutions included.

Despite the growing interest of the relevant governmental institutions in the field of RDs in the recent period and some positive prospects, issues regarding institutional management, vertical and horizontal organization, and long-term funding in the field of RDs still remain largely unresolved. Since the effective management of RDs is one of the crucial measures for successfully dealing with this important public health problem, the introduction of promising campaigns, such as an ecosystemic approach, should receive further attention and governmental impetus.

#### 3.1.3. Digitalization and Data Management Aspects

Although a basic digitalization of the Slovenian healthcare system was established relatively early, the existing IT infrastructure is still rather heterogeneous, in the sense of both degrees of the digitalization of particular healthcare fields and/or services and the numerous different IT solutions. Healthcare providers in individual fields have been struggling with certain issues concerning the establishment of dedicated institutional and national IT solutions. In this respect, the distinct field of RDs finds itself in a difficult position, as it does not have any specialized IT solutions for local outpatient work or central IT platforms to collect, analyze, and display data on RDs at the national level—such as the National RD Registry [8]. The planned establishment of the National RD Registry is crucial to this end, as emphasized by the focus group participants. With the successful introduction of eHealth solutions in previous years and the use of uniform infrastructure and data exchange protocols, interoperability issues have been largely eliminated. However, in order to overcome the potential problems associated with interoperability between the local healthcare information systems and the IT solutions in the RD ecosystem (i.e., the planned National RD Registry and other IT platforms), adequately structured and standardized healthcare data should be used in data transfers [24,25]. Data collection, reporting, and usage should be based on common principles such as findable, accessible, interoperable, and reusable (FAIR) data and information [26]. In the context of the potential introduction of an ecosystemic approach, special attention should be afforded to the core eHealth solution, named the Central Registry of Patient Data (CRPD). The CRPD contains a summary of patients’ healthcare data and medical records extracted from different data sources (also from the planned National RD Registry). The CRPD allows healthcare providers in Slovenia to access and exchange healthcare data to ensure high-quality patient treatment, and is of great importance for the management of the entire healthcare system. Accordingly, the CRPD should be extensively utilized for various uses in the RD ecosystem (planning and monitoring healthcare treatment, analysis, and decision-making; epidemiological and other public health studies; the preparation of public healthcare policies/programs).

Generally, IT solutions should facilitate easy but safe interaction between all national healthcare providers from prenatal to adult medicine in cases involving the diagnosis of an RD. The planned National RD Registry may provide the core element of patient identification via a diagnosis and related clinical data. The content in the expected National RD Registry may encourage optimal follow ups, with an individualized medical plan and through a responsible healthcare team, aided by the IT-enabled ecosystem. The National RD Registry is expected to serve as the main source for international data exchange. The compatibility of the IT solutions is required in order to safely share patient data, images, and video data with specialized international medical centers for consultations. The NCP could become a central hub for healthcare providers and patients. However, presently, staff and data resources are insufficient, since NCP activities are not adequately funded or professionally supported. Consequently, the general expectations of patients with RDs are not fulfilled. The IT-enabled ecosystem is expected to connect a broad pool of professional collaborators, including the social sector, employment agencies, educational institutions, and legal services with the larger, organized, and proficient team of the NCP.

During a cross section of the critical aspects in the field, the focus group participants highlighted the questions that should be sensibly addressed by the ecosystemic approach in order to facilitate a functional arrangement for the management of RDs in Slovenia. Although there were initially some discussions and dilemmas on the content of certain issues raised, the final list of questions presented below ultimately included all the relevant concerns highlighted by the participating experts (Table 3).

### 3.2. Outlining an IT-Enabled Ecosystemic Approach

**Figure 1:**
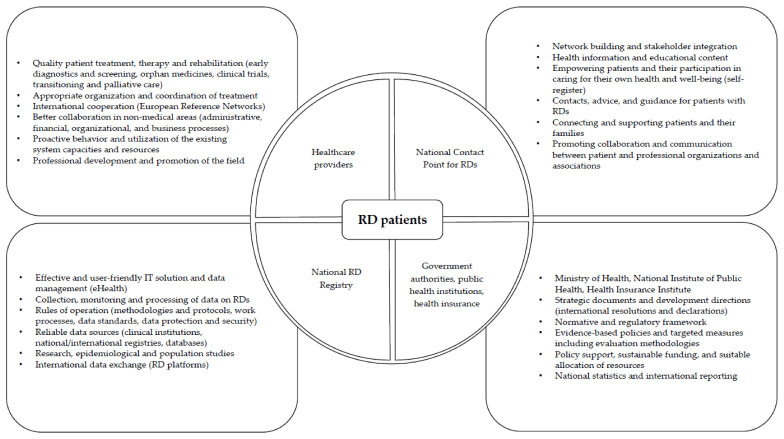
An outline of the proposed IT-enabled RD ecosystem in Slovenia.

Arising from the analysis of the current situation in the field of RDs and predominantly the needs of the stakeholders, we hypothesized an outline of the preferred RD ecosystem in Slovenia (Figure 1) based on the recommendations of the focus group participants and a literature review. The planned RD ecosystem in Slovenia should aim to involve all relevant stakeholders, especially healthcare institutions (including members of the European Reference Networks—ERNs), patients and professional associations (including the umbrella RD patient organization), the National Institute of Public Health, the Ministry of Health, the Health Insurance Institute of Slovenia (HIIS), and international and supranational entities. Additionally, the desired ecosystem should harmonize the relationships that exist between them, complement organizational arrangements, align business processes, and provide user-friendly and effective IT tools and platforms, especially the National RD Registry and the NCP for RDs. All operations of the constituent entities and mechanisms required for the functioning of the ecosystem must be supported by feasible sector policies and strategic documents, as well as a normative and regulatory framework and sustainable funding. The essential components of the proposed RD ecosystem and their functions are presented in the following sections.

#### 3.2.1. The National RD Registry

The recommended establishment of the National RD Registry is believed to be one of the essential components of the RD ecosystem, thus providing a means for monitoring and managing RDs in Slovenia and consequently improving the treatment of patients with RDs [8,27]. The National RD Registry represents a central tool for the collection and processing of all data on RDs, clinical research, and population studies, and may significantly improve access to the relevant data by all stakeholders involved in the RD ecosystem [25,26]. Moreover, RD registries facilitate comprehensive surveillance of the prevalence and incidence of RDs, the observation of other relevant parameters (diagnostic features, natural histories, therapies and treatments performed, outcomes), and allows for a well-founded evaluation of different aspects of healthcare procedures and outcomes. Quality RD registries *ipso facto* provide a beneficial and applicable platform in all stages of evidence-informed healthcare policymaking, including economic evaluations, and may contribute to a significant advancement in the management of RDs in general [28]. There is an ongoing discussion about whether the possibility of developing a drug for a particular disease increases if well implemented registries and active patient organizations exist [29,30,31].

Therefore, the development of RD registries is one of the EU’s priorities in the field of monitoring and controlling RDs, as evidenced by specific recommendations and measures implemented to support the development of such registries in different EU health resolutions, strategic documents, and EU-funded projects [32]. In accordance with the recommendations and initiatives, several EU countries (France, Spain, Italy, Slovakia, Belgium, Bulgaria, and Sweden) have been working intensively on the development and implementation of RD registries [26]. In view of this, the Slovenian National RD Registry should be able to connect with international RD registries, including the European platform on RD registration [33].

The secure reporting system and data flows included in the National RD Registry must be based on clearly defined business processes and disciplined data processing at their source. At the same time, all data inputs must have a standard format and their transfer must be logically and physically controlled according to the methodological guidelines and rules of operation. All security and personal data protection aspects concerning the National RD Registry and other IT solutions in the RD ecosystem should be in line with national legislation and best security practices.

#### 3.2.2. NCP for RDs 

The Ministry of Health initiated a project governed by the UMCL Division of Pediatrics to establish the NCP for RDs in 2015. The NCP was successfully established in 2016 [34]. The aim of the NCP is to create a network of stakeholders and to provide patients, their families, and experts with access to high-quality information on the treatment of RDs is Slovenia. The long-term objective, focused on raising awareness of RDs, looks to offer patients the option to self-register through the NCP.

The RD policy encourages the active engagement of people living with RDs or their family members in shaping the decisions concerning their care. Patients’ empowerment increases their capacity to express their needs and concerns, but it also encourages their involvement in processes that help people to reclaim control over their own lives. RD helplines are an important service and provide quality information and support [35]. The Slovenian NCP aims to support patients with RDs in different ways, such as through daily e-mail and phone contact, as well as a Facebook profile page and blog in the Slovene language, which is valuable as it makes the information accessible to people who do not, on average, have an advanced level of English. The official coordinator of the NCP is a medical nurse, permanently employed in a part time position. The physicians in the professional NCP team work as volunteers and they share medical fields involving different areas of expertise. They help in revising medical information about RDs on the website and provide answers to inquiries from different callers and visitors to the NCP.

The NCP team has faced many challenges. The expectations of the callers are diverse, and their questions often demand broad medical expertise. Patients’ questions and requests that are addressed to the NCP are occasionally very detailed and concern complex personal health conditions. The NCP team aims to fulfill caller expectations, but the group of undiagnosed patients with suspected RDs represents a particularly fragile subpopulation. They are provided with basic information and directed to the relevant specialists in the national healthcare system or consulted with regard to the possibility of receiving a second opinion. Another pressing challenge is the transition of patients with RDs from pediatric to adult healthcare. The role of the NCP is very important in this process, as it provides connections between medical professionals and informs patients about possible solutions.

#### 3.2.3. Health Insurance and Orphan Medicines for RDs

The relatively small size of Slovenia and its unified public health system under one financier—the HIIS—constitute advantages in treating RD patients. Specialized centers for specific groups of RDs have operated in Slovenia for several years, managing the large demand for RD management at only a few locations. RDs are treated in the same way as all other diseases. The Health Care and Health Insurance Act [36] does not classify RDs and financing as different to the treatment and financing of other diseases. According to the special provisions of the Act, most RDs belong to the groups of diseases that are 100% financed from compulsory health insurance.

Marketing approval by the European Medicine Agency was granted to 164 orphan medicines for RDs, whereas 28 medicinal products are available for the treatment of hereditary metabolic diseases [37]. There are currently 73 orphan medicines registered in Slovenia. In 2019, ten new orphan medicines were newly listed, and in February 2020 an additional five were listed [38].

#### 3.2.4. ERNs and Patient Organizations for RDs

The year 2017 saw the establishment of 24 ERNs, which represent virtual institutional networks connecting healthcare providers across Europe, with the aim of treating complex or rare diseases that require highly specialized treatment and the aggregation of knowledge and resources. Slovenia has been very successful in joining ERNs. In 2017, nine healthcare providers from Slovenia joined ERNs as full members. In 2019, another seven providers joined ERNs as associate national centers, along with one other provider, namely, the Clinical Institute of Genomic Medicine, as the Slovenian national hub for ERNs. Slovenian healthcare providers currently cooperate with all 24 ERNs. This places Slovenia among the top countries in terms of cooperation with ERNs, as only ten European countries, including Slovenia, participate in all 24 ERNs [38].

Patients organizations act as key partners and play an important role in the co-creation of ERNs. They work and help in collaborative efforts to achieve better accessibility, diagnosis, and treatment. Accordingly, ERN representatives and affiliates can participate in virtual consultations with the board of medical experts to discuss diagnostic procedures and the treatment of patients [39]. However, the appropriate level of funding is an essential prerequisite for professional work concerning data management (including registries), virtual consultations, clinical practical guidelines, education, training, and research in ERNs [40]. Additionally, the healthcare systems of Slovenia and other ERN members does not provide financial incentives for national reference centers for RDs. The European Commission continues to encourage the Ministries of Health of EU Member States and hospital managers to allocate working time, additional funds, and administrative support once a hospital becomes a member of an ERN or an affiliated partner.

In Slovenia, there are more than 30 RD patient associations or initiatives in the field of RDs [30], including an umbrella organization (the Association of Patients with RDs of Slovenia) [41]. Within these patient associations, patients can find support, exchange experiences and additional practical knowledge about their disease, and find answers to their social and medical concerns. Some of these associations are members of Eurordis, a European alliance of 917 RD patient organizations. RD patient organizations thus possess considerable social and intellectual capital, including a wide and well-functioning network of collaborators and volunteers, which is indispensable for providing patient support and ensuring the efficient functioning of the RD ecosystem [42].

#### 3.2.5. Early and Specialized Diagnostics of RDs

An important development in 2018 was the introduction of additional newborn screening (NBS) tests for an additional 17 inborn disorders at the University Children’s Hospital at UMCL (hitherto, there had been two), which are routinely performed on newborns and are financed through compulsory health insurance [43]. The implemented NBS program employs next-generation sequencing as a confirmatory method [44]. We expect to expand the program further and include other relevant RDs soon. In the long-term, we expect that genomic testing will be gradually implemented as a first-line NBS test, allowing for the detection of a wide range of RDs.

Among the dedicated institutions, the Center for Undiagnosed RDs is worth mentioning, which works in the framework of the Clinical Institute of Genomic Medicine at UMCL. It represents the first regional specialized unit for the diagnoses of undiagnosed genetic RDs. Specific laboratory services are performed in the laboratories of KOPA Golnik, UMC Ljubljana, and UMC Maribor. Funds for the diagnosis and medical treatment of RDs, and diagnostic tests of tissue and blood samples at the listed clinics and institutes are provided through the HIIS, for use in tertiary medical treatment. 

#### 3.2.6. Transitioning Care and Palliative Care in RDs

The care of adult patients with RDs that are mainly diagnosed at a pediatric age (e.g., inborn errors of metabolism) has become one of the major challenges for healthcare providers, as recognized by the ERNs [45]. Several specialized adult care centers were recently established in Slovenia with a plan to gradually develop multidisciplinary teams of experts taking care of adult RD patients in their respective fields. One such recent example is the Center for Adult Patients with Inborn Errors of Metabolism at the UMCL, established in 2018. 

For the RDs with a poor prognosis, palliative care should be planned in advance and should adequately address the needs of families [46]. Recently, a multidisciplinary team for pediatric palliative care was established at the University Children’s Hospital at UMCL, which will also care for pediatric patients with RDs across the country.

## 4. Discussion

Even though the research shows that RDs represent a serious public health concern [1,2,6,15,16,28], the general lack of epidemiological data on numerous RDs makes it impossible to calculate their actual burden [47]. According to some estimates, the number of patients with an RD in Slovenia exceeds the number of patients with type 2 diabetes [1]. As is the case elsewhere, patients with RDs typically face late diagnosis, long-lasting and expensive treatment (if medication is even available), and social consequences [7,31]. For individuals affected by these largely unpreventable diseases, a comprehensive public health approach, comprising the establishment of an operative RD ecosystem, would provide an important step towards more coherent and effective medical treatment, and significant improvements in the entire field of RDs [8,10]. Raising awareness of RDs and the multilateral efforts made to regulate the field can significantly contribute to a more equitable allocation of resources, which, in its current form, is inadequate with regard to the scope of the problem and in comparison with some other diseases deemed to be public health priorities [2].

The Slovenian healthcare system operates relatively well in the field of RDs, given the available resources and other systemic circumstances mentioned above. The current systemic arrangement in the field of RDs is a hybrid structure that combines both obsolete (policy, institutional, and organizational aspects) and modern components (treatment approaches, screening, patient support).

The existing system attempts to adapt to major innovations in the field of RDs, be they novel treatments (orphan medicines), new diagnostic and epidemiological tools (the NCP for RDs), new therapeutics, or the active participation in ERNs. Nevertheless, there are considerable opportunities for progress in the field of RDs, and the existing constraints will need to be addressed as comprehensively as possible in the future. This study facilitated a methodological examination of the existing state of affairs in the field of RDs in Slovenia, while the focus groups assisted in the identification of the most important systemic limitations and challenges, which predominantly touch upon the following issues:

- The absence of an up-to-date policy framework, strategic documents, action plan, and evaluation metrics, including public health objectives;
- Normative deficiencies and systemic/institutional non-compliance with regulations in the field;
- Non-optimal institutional collaboration and coordination, and rather unsettled organizational and processual matters;
- A substantial lack of resources (material, human, IT, organizational);
- Inconsistent clinical and institutional practice in certain segments of the coding procedures; and
- Isolated, non-user-friendly, and ineffective IT tools and platforms in the entire field of RDs.

The challenges identified have a wide-ranging adverse effect on the critical factors and hamper efforts to implement improvements throughout the field of RDs. Forthcoming strategies and measures will need to resolve these issues, since they could have a decisive impact on the establishment of an RD ecosystem and inhibit further developments that are urgently required, in the field of RDs. In view of these factors, there is compelling evidence from other social subsystems and industries supporting the idea that the establishment of an RD ecosystem would provide a foundation for the systemic regulation of RDs in the country [8,10,11]. Every component outlined in the draft has a specific role in the proposed ecosystem constellation and should provide a significant contribution to the overall functioning of the RD ecosystem and offer benefits to either internal (professional) or external (patients) users. It is important to note that some charted components perform a more important role than others due to their integrative nature. Namely, components such as the National RD Registry, the NCP, health insurance, the treatment of RDs, international cooperation, and patient organizations are cross-sectorial and would ensure the implementation of appropriate work processes throughout the RD ecosystem itself, thus connecting all constituent components and stakeholders, especially healthcare professionals and patients.

Based on the literature review and focus group findings, a well-organized RD ecosystem, including its operative constituent components, could substantially contribute to a more comprehensive monitoring of RDs [25], improved and better coordinated patient treatments [11], reduced inequality, and to an increasingly effective mode of two-way communication between RD patients and other stakeholders [48]. Furthermore, focusing on the policy, financial, and development aspects, an IT-enabled RD ecosystem could provide significant benefits for all healthcare decision-makers by enhancing evidence-informed policymaking, providing a convenient platform for the approximation and allocation of the required resources, and facilitating business process re-engineering, organizational restructuring [9], and technological innovations, including predictive analytics and artificial intelligence [49]. The content analysis process and the following inferences revealed that the institution of an inclusive RD ecosystem necessitates in-depth reforms of the existing arrangements, including the commitment of the stakeholders, sustained by focused policy measures and appropriate subsidies [50,51,52].

The conceptualization of the ecosystemic approach, derived from the content analysis, has proved challenging, as it was crucial to consider all the specifics of the healthcare system, equally and in regulatory terms (policy, strategic) as well as institutional (organizational, process) and IT terms [53,54]. Hence, the -requirements identified for the integration of the outlined components and potential establishment of an IT-enabled RD ecosystem will have to be grounded in a feasible strategy that accurately delineates the organizational structure, processes, roles, assignments, and competences of each constituent component. Due to the complexity and significance of the components involved, an RD ecosystem would inevitably require efficient collaboration, coordination, and process orientation, and an unwavering focus on the success of patient treatment and their general well-being. All these efforts will have to be supported by substantial levels of funding and the support of healthcare authorities. The proposed ecosystemic approach does not attempt to impose a simplified “one-size-fits-all” model for the resolution of the numerous concerns associated with the management of RDs. The study offers a valuable analysis of the contextual settings in the field of RDs in Slovenia, and could provide an applicable platform for upcoming experiments and developments in this domain.

### Methodological Limitations and Forthcoming Research Orientations

The applied research framework contains an inherent methodological limitation. Namely, as suggested earlier in the text, current healthcare arrangements in Slovenia do not encompass a clearly delineated and adequately organized RD ecosystem, and it was therefore necessary to partially predict the effects of the RD ecosystem on the basis of theoretical ideas, a literature review, and suggestions provided by the focus group participants. Accordingly, the overall characteristics and implications of the proposed RD ecosystem have been hypothesized and gauged without conducting trials in real RD settings. These issues may therefore raise certain methodological dilemmas; hence, the research conclusions could be subject to different interpretations. Future studies should focus on an in-depth investigation of RD ecosystem implications for healthcare system performance in the field of RDs, and particularly on the simulation and testing of RD ecosystem features in an actual healthcare environment. Further inquiries should primarily attempt to define critical success factors and focus on formulating workable recommendations for the establishment of similar RD ecosystems in countries worldwide, since this field is generally under-regulated and underdeveloped, and, consequently, patients still do not receive ample medical treatment in many places due to non-medical reasons and dysfunctional systems in the field of RDs. Despite certain methodological dilemmas, the study reveals the complex subtleties and multifaceted concerns in the field of RDs in Slovenia, offering a clear impression of the critical role of an IT-enabled RD ecosystem, hopefully contributing to further developments in this fields.

## 5. Conclusions

The problems concerning the management of RDs in Slovenia largely overlap with the common challenges, outlined by the study, experienced on a global scale. In view of this, the establishment of an IT-enabled RD ecosystem in Slovenia would provide advantages for patients and healthcare professionals, on one hand, and healthcare managers and decision-makers, on the other. However, it is clear that introducing an ecosystemic approach necessitates the alteration of the entire operational paradigm in the field, including the adoption of sensible healthcare policies and a strategic framework. This should be followed by the extensive transformation of the constituent components so that they comply with the institutional, organizational, processual, and IT requirements of a functional RD ecosystem.

The establishment of an RD ecosystem in Slovenia would have significant potential and could be a determining factor in the future development of the field. Nevertheless, if the developed RD ecosystem has an uncoordinated and hollow structure, even if properly designed, it will not considerably improve the odds of patients with RDs. Ensuring the delivery of effective healthcare in this specific field is dependent on patient-centeredness, and responsiveness to patients’ needs. The successful implementation of an RD ecosystem would lead to the formation of a network of stakeholders that could actively participate in the further development of integrated and effective patient care. In this way, we could overcome various challenges and numerous systemic inconsistencies, which represent the main obstacles to the long-term and comprehensive regulation of RDs in Slovenia.

## Figures and Tables

**Table 1:** Structure and characteristics of the sample.

Institution	Number of Participating Experts	Areas of Work and Research
University Medical Center Ljubljana (UMCL)/University of Ljubljana, Faculty of Medicine	11	Pediatric and adult endocrinology and diabetology, pediatric immunology, epidemiology, molecular genetics, inherited metabolic diseases, clinical genetics, human reproduction, neurogenetics, dysmorphology, neurology, pharmacy, biochemistry and molecular biology, clinical biochemistry, laboratory medicine
National Institute of Public Health	3	Public health, the healthcare system, healthcare system organization and resources, health insurance and reimbursements, healthcare data, data management, methodologies, statistics, and data analytics
University Medical Center Maribor/University of Maribor, Faculty of Medicine	6	Microbiology and immunology, human reproduction, medical genetics and cytogenetics, biochemistry and molecular biology, embryogenesis and dysmorphology, blood cancer, atherosclerosis and rare cardiovascular diseases, molecular genetics, molecular oncology
Slovenj Gradec General Hospital	2	The cardiovascular system, metabolic and hormonal disorders
IT companies	2	Systems and cybernetics, computer science and informatics, IT architecture, electronic health records, registries, health information systems, IT tools, web apps and platforms for patients, IT system infrastructure, designing and building IT solutions, data standards, data security and privacy (according to the General Data Protection Regulation (GDPR) and safe-by-design principle)

**Table 2:** Polling results on the identification of the critical aspects in the field of RDs.

Critical Aspects	Position of the Focus Group Participants
Policy and normative aspects	24/24 respondents agreed
Institutional and organizational aspects	24/24 respondents agreed
Digitalization and data management aspects	24/24 respondents agreed

**Table 3:** The baseline questions to be addressed by the ecosystemic approach.

	Questions Identified by the Focus Group Participants
Q1	How is the strategic (policy) and regulatory framework currently regulated and does it offer all the necessary bases for further development in the field of RDs?
Q2	Who are the main stakeholders in the field, what are their roles, and what competences and powers should they have?
Q3	What should the starting points be for the preparation of the relevant strategic and development orientations, including sectoral policies, operational measures, and the evaluation framework?
Q4	What actions should be taken by the government to ensure continuous development in the field and the best patient treatment under the given circumstances?
Q5	What are the main benefits regarding the treatment of patients that we want to ensure by introducing an IT-enabled ecosystemic approach in the field of RDs in Slovenia?
Q6	How to facilitate the appropriate arrangement and mobilization of all necessary experts and resources in Slovenia and abroad for the treatment of patients with RDs?
Q7	What activities and systemic measures are additionally needed by healthcare providers for more efficient management of RDs in Slovenia?
Q8	How to ensure the use of available systemic and institutional potentials to improve patient treatment and enable more effective management of RDs?
Q9	How to establish a comprehensive IT infrastructure that will connect stakeholders and provide adequate support for working with patients?
Q10	Which local or national IT solutions need to be established and which functionalities and operations should these IT solutions enable?
Q11	What procedural, methodological, organizational and other preconditions must be put in place for the successful use of the potentials offered by IT solutions?
Q12	What conditions must the designed IT solutions meet and what goals should they serve in terms of the secondary use of data and international cooperation?
Q13	What information and services should the NCP provide to patients (and their families) on a personal level and what information and services to the general public?
Q14	What information and services should the NCP provide to healthcare professionals and what information and services to patient associations and government institutions?
Q15	What are the longstanding goals of the NCP in terms of its development and use?
